# Complete Genome Sequence of Strain SS-5, a Magnetotactic Gammaproteobacterium Isolated from the Salton Sea, a Shallow, Saline, Endorheic Rift Lake Located on the San Andreas Fault in California

**DOI:** 10.1128/MRA.00928-20

**Published:** 2021-01-07

**Authors:** Denis Trubitsyn, Caroline L. Monteil, Corey Geurink, Viviana Morillo-Lopez, Luiz Gonzaga Paula de Almeida, Ana Tereza Ribeiro de Vasconcelos, Fernanda Abreu, Dennis A. Bazylinski, Christopher T. Lefevre

**Affiliations:** a Department of Biological and Environmental Sciences, Longwood University, Farmville, Virginia, USA; b Aix-Marseille University, CNRS, CEA, UMR7265 Biosciences and Biotechnologies Institute of Aix-Marseille, Saint Paul lez Durance, France; c School of Life Sciences, University of Nevada Las Vegas, Las Vegas, Nevada, USA; d Laboratório Nacional de Computação Científica, Petrópolis, Brazil; e Instituto de Microbiologia Paulo de Goes, Universidade Federal do Rio de Janeiro-UFRJ, Rio de Janeiro, Brazil; University of Southern California

## Abstract

We report the 3.7-Mb genome sequence of strain SS-5, a magnetotactic, sulfur-oxidizing rod and member of the family *Chromatiaceae* of the class *Gammaproteobacteria*, which biomineralizes membrane-bound, elongated, prismatic octahedral, magnetite nanocrystals. This genome sequence brings further diversity for understanding the origin and evolution of magnetotaxis and magnetosome biomineralization.

## Abstract (precis)

We report the 3.7-Mb genome sequence of strain SS-5, a magnetotactic, sulfur-oxidizing rod and member of the family *Chromatiaceae* of the class *Gammaproteobacteria*, which biomineralizes membrane-bounded, elongated, prismatic octahedral, magnetite nanocrystals. This genome sequence brings further diversity for understanding the origin and evolution of magnetotaxis and magnetosome biomineralization.

## ANNOUNCEMENT

Magnetotactic bacteria (MTB) biomineralize membrane-bound magnetic nanocrystals to form bacterial organelles, the so-called magnetosomes (1). The formation and arrangement of magnetosomes within these cells are genetically controlled (2, 3). The magnetosome chain allows MTB to passively align along geomagnetic field lines as they swim (4). MTB are not represented in a well-defined taxonomic group of prokaryotes but, instead, are distributed over several bacterial taxa (5, 6).

Here, we report the genome sequence of the magnetotactic gammaproteobacterium strain SS-5, isolated from a sediment sample of the Salton Sea in California (7). DNA was extracted from a 2-liter pure culture of cells grown autotrophically (7) using the standard phenol-chloroform assay. All kits were used according to the manufacturers’ standard recommendations. A Clean & Concentrator (Zymo Research) kit was used for DNA purification. DNA quality was confirmed with the Agilent Bioanalyzer system, and samples were quantified with a Qubit v2.0 fluorometer. Libraries were prepared according to the manufacturer’s standard protocol using a GS FLX Titanium 3-kb span paired-end library kit. 454 genome sequences were obtained in one run on the GS FLX system sequencer (Roche) with average read lengths of 216 bp. Sequence duplicates were removed using the cd-hit v4.6.1 algorithm (8). All remaining 595,403 reads (234,621,138 bp, 63× coverage) were used for assembly. The assembly performed with Newbler v2.6 (Roche) (9) using default parameters resulted in 9 scaffolds (*N*_50_, 1,196,217 bp). Gap closure was completed using a previously published procedure (10). The genome was rotated, and the *dnaA* gene was positioned close to the beginning of the consensus sequence. The completed genome of SS-5 was annotated using PGAP (11) and consists of 3,729,439 bp with a G+C content of 61.6%: 3,223 predicted coding DNA sequences, 51 tRNAs, and two identical sets of 5S/16S/23S rRNAs.

Strain SS-5 is a member of the *Gammaproteobacteria* that was originally determined to belong to the order *Chromatiales* (7). The average amino acid identity (two-way AAI) between SS-5 and its closest neighbors (identified by phylogenetic reconstruction of all available genomes of *Gammaproteobacteria* type strains) was estimated with the enveomics collection toolbox using default parameters (12). SS-5 did not get a higher AAI value than 55.67% with other *Chromatiaceae* members (the best score was with Allochromatium vinosum DSM 180; GenBank accession number NC_013851). As a newly identified member of the *Gammaproteobacteria*, this strain will provide a valuable resource for further characterizing the metabolic potential of this group. For instance, the genes necessary for magnetosome formation were found to be present in the genome of SS-5 (13). Thus, this closed genome will provide the opportunity to further elucidate the origin and evolution of magnetotaxis.

### Data availability.

The SS-5 genome sequence has been deposited in GenBank under BioProject number PRJNA491989 and BioSample number SAMN10093509. The genomic raw sequencing reads are available in the Sequence Read Archive (SRA) database under accession number CP032508.1.
